# An Automatic Calibration Method for the Field of View Aberration in a Risley-Prism-Based Image Sensor

**DOI:** 10.3390/s23187777

**Published:** 2023-09-09

**Authors:** Zhonglin Lin, Wenchao Liu, Jinyu Gan, Jilian Lu, Feng Huang, Xianyu Wu, Weixiong Wang

**Affiliations:** School of Mechanical Engineering and Automation, Fuzhou University, Fuzhou 350108, China; linzhonglin@fzu.edu.cn (Z.L.); 210220065@fzu.edu.cn (W.L.); 210227077@fzu.edu.cn (J.G.); lujilianfzu@163.com (J.L.); xwu@fzu.edu.cn (X.W.); weixiongfzu@163.com (W.W.)

**Keywords:** risley prisms, field of view, aberration correction, automatic calibration

## Abstract

Risley-prism-based image sensors can expand the imaging field of view through beam control. The larger the top angle of the prism, the higher the magnification of the field of view, but at the same time, it aggravates the problem of imaging aberrations, which also puts higher requirements on the aberration correction method for the Risley-prism-based image sensor. To improve the speed, accuracy, and stability of the aberration correction process, an automatic calibration method for the Risley-prism-based image sensor is proposed based on a two-axis turntable. The image datasets of the calibration plate with different prism rotation angles and object distances are acquired using a two-axis turntable. Then, the images of the calibration plate are pre-processed using the bicubic interpolation algorithm. The calibration parameters are finally calculated, and parameter optimization is performed. The experimental results verify the feasibility of this automated calibration method. The reprojection error of the calibration is within 0.26 pixels when the distance of the imaging sensor is 3.6 m from the object, and the fine aberration correction results are observed.

## 1. Introduction

The Risley prism, which Rosell first developed in 1960 as a prism scanner [1], comprises two wedge prisms that are aligned and may be rotated. Other Risley prism devices may consist of a pair of tilting, rotational and tilting, or translational prisms [2,3,4]. By altering the angle of the prism, the Risley-prism-based image sensor realizes the deflection of the optic axis, which has the effect of enlarging the imaging field of view (FOV). The Risley-prism-based imaging sensor has a wide variety of applications in wide-area target search, recognition, tracking imaging, and laser scanning as a result of the ongoing advancement of related research [5,6,7].

In the fields of microscopic observation, traditional microscopes with a small FOV and a fixed optic axis do not provide enough visual information for multi-target and dynamic target observation. Currently, many studies use Risley prisms as an optic axis scanning mechanism, resulting in a compact optical scanning microscope system with a large imaging FOV and high resolution, which can be used for microscopic observation in biomedical, microelectromechanical, and other fields [8]. Warger, W. C. et al. designed a confocal microscope device incorporating the Risley-prism-based imaging sensor for the detection of skin lesions [9,10]. Li, G. et al. [11] demonstrated a novel scanning mechanism in dual-axes confocal endomicroscopy to visualize molecular expression in the epithelium. Shirazi, A. et al. [12] proposed a side-view dual-axes confocal endomicroscope that can be inserted repetitively in hollow organs of genetically engineered mice for in vivo real-time imaging in horizontal and vertical planes. Bravo-Medina, B. et al. [13] proposed a novel technique using Risley prisms for area-scan with ballistic photons. Tao, X. et al. [14] designed a variable-viewing angle imaging system by combining a scanner and the Risley-prisms structure, which can be used to observe dynamic targets in the microscope. In the field of ophthalmology, the Risley-prism-based imaging sensor can be utilized for binocular accommodation measurements [15,16,17], i.e., by rotating a pair of optical wedge prisms with a small apex angle, the visual axes of both eyes are altered to achieve scanning of the visual axes in a small angular range. The Risley-prism-based imaging sensor can also be used in optical coherence tomography (OCT) to realize scanning imaging, and the small size of the structure facilitates the construction of a compact OCT system [18,19,20].

In robotic vision tracking, Souvestre, F. et al. [21] used the Risley-prism-based imaging sensor to dynamically deflect the laser beam to realize overall scanning and to expand the scanning field to realize multi-target laser tracking. Li, A. et al. [22] proposed a visual tracing model based on Risley prisms and two real-time visual tracing strategies, which effectively avoid the problems of target loss and tracking instability. Zhou, Y. et al. [23] avoided the control singularity at the crossing center path by studying the FOV of the Risley prism system.

In these imaging applications, the use of the Risley-prism-based imaging sensor will inevitably introduce additional imaging chromatic aberrations and distortion aberrations. The chromatic aberrations caused by Risley prisms have been intensively investigated by many researchers. By designing diffractive optics or gluing prism materials with different refractive coefficients and dispersion parameters, the imaging chromatic aberrations can be effectively corrected [24,25,26].

Since the angles of light incident on the prisms in the objective FOV are not uniform, it is impossible to obtain a consistent deflection of the light from the entire FOV when rotating the two prisms to move the imaging view axis to a given position, which eventually produces imaging aberrations. For smaller angular deflections, the imaging aberrations are not obvious. Nevertheless, when the deflection angle of the view axis is large, the imaging aberrations caused by the prism become particularly significant and can eventually lead to unrecognizable imaging targets. The imaging aberrations caused by prisms are different from lens aberrations. Lens aberrations are aberrations caused by the camera lens and can be effectively corrected by a mature calibration model [27,28,29]. For the imaging aberrations caused by the Risley prisms, there is no complete and mature model to characterize, and traditional aberration correction methods cannot be used to correct such aberrations. Lavigne, V. and Ricard, B. [30] proposed a single-image mapping transformation correction method for imaging mosaic stitching applications. This transform is a linear correction method that enables fast aberration correction in real-time. However, this method only involves the mapping transformation of image pixel points without considering the actual propagation and imaging of light in the system, and its aberration correction accuracy is not high. Zhou, Y. et al. [31] established a spatial refraction model of the Risley-prism sensor and used the inverse ray tracing method to achieve aberration correction. The correction effect of this method is better, but this correction method is nonlinear, and the correction speed is slow. Qi, Y. et al. [32] used multiple camera pre-calibration, the establishment of a calibration database, and an interpolation reprojection algorithm based on the table look-up method to achieve aberration correction in order to improve the correction quality and speed. The above methods require a large amount of calibration plate material in the pre-calibration process, and the manual acquisition process is tedious and has too many chance factors, which makes it difficult to guarantee its accuracy and stability and also requires a lot of time; all these defects limit the promotion of dual prism imaging sensors to some extent.

To address these problems, an automatic calibration method is proposed for the Risley-prism-based imaging sensor based on a high-precision two-axis turntable. We study the main factors affecting the calibration error of the Risley-prism-based imaging sensor and propose an image pre-processing method to solve the problem of low resolution in special areas of the imaging FOV. In this paper, the Risley-prism-based imaging sensor is fixed on a two-axis turntable, and the turntable is rotated according to a certain rule to obtain the original calibration images with a specific spatial distribution, and then the calibration is performed automatically. It is proved that this method not only improves the efficiency and stability of calibration but also obtains high accuracy of aberration correction.

The innovations of this paper are as follows:

- Designing a Risley-prism-based imaging sensor based on a two-axis turntable to take a large number of calibration plate images and provide a dataset for the subsequent calibration.
- Proposing an automatic calibration method for the FOV aberration in a Risley-prism-based image sensor. The calibration process is completed quickly and accurately through the automatic control of the turntable and Risley prism.
- Adopting the bicubic interpolation algorithm to pre-process the images, which solves the problem of the failure of corner detection in the special FOV caused by the low resolution, and the reprojection error of the calibration results meets the requirements of FOV aberration correction.

The remainder of this paper is organized as follows: the second part carefully analyzes the imaging principle of the Risley-prism-based imaging sensor and elaborates on the methods of image pre-processing and aberration correction; the third part describes the structural design and hardware system design of the Risley-prism-based imaging sensor; the fourth part introduces the detailed procedure of the calibration method of the Risley-prism-based imaging sensor based on the two-axis turntable; the fifth part designs experiments to verify the feasibility of the automated calibration method of the Risley-prism-based imaging sensor; the last part summarizes this study.

## 2. Theoretical Model of the Risley-Prism-Based Imaging Sensor

**Figure 1:**
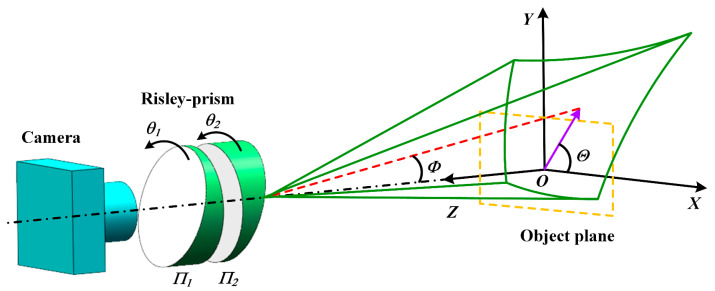
The principle of the typical Risley-prism-based imaging sensor.

In order to investigate the main factors affecting the calibration error of the Risley-prism-based imaging sensor, the principle of the typical Risley-prism-based imaging sensor [33] is shown in Figure 1, which consists of a fixed camera and a pair of prisms *Π*_1_ and *Π*_2_ that can be rotated around the *Z*-axis, respectively. The rotation angles of the two prisms are $\theta_{1}$ and $\theta_{2}$. The wedge angles of the two prisms are $\alpha_{1}$ and $\alpha_{2}$, and the refractive indices are $n_{1}$ and $n_{2}$. The initial main cross-section of the two prisms lies in the *XOZ* plane, with the thickest end of $\Pi_{1}$ pointing in the negative direction of the *X*-axis and the thickest end of $\Pi_{2}$ pointing in the positive direction of the *X*-axis. The combination of the deflection angle of the outgoing light Φ and the azimuth angle *Θ* can represent the deflection effect of the Risley-prism system on FOV. Based on the rotation of each of the two prisms, the view axis of the camera (red dashed line) moves within a specific cone, thus changing the imaging FOV. At the same time, the original rectangular FOV (yellow dashed line) is pulled by the prisms into an irregular FOV (green solid line).

### 2.1. Beam Tracing Model of the System

According to the pinhole model of the camera [34,35], the perspective projection transformation of the incident light vector $\hat{S}=[x,y,z{]}^{T}$ of the Risley prism to the aberration-free ideal point $\left(x_{h},y_{v}\right)$ of the camera image plane can be expressed as:

(1) $$\{\begin{array}{l} {\left(x,y,z\right)}^{T}={\left(x_{h},y_{v},f\right)}^{T}\\ x\in [-f\tan\frac{h}{2},f\tan\frac{h}{2}]\\ y\in [-f\tan\frac{v}{2},f\tan\frac{v}{2}] \end{array},$$

where *h* denotes the horizontal FOV of the camera, *v* denotes the vertical FOV, and *f* denotes the focal length.

**Figure 2:**
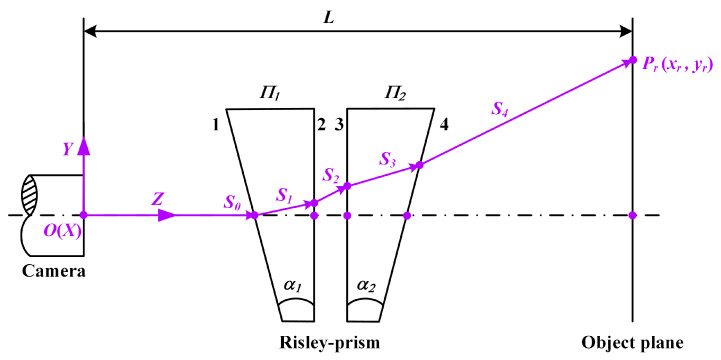
Beam propagation process in the Risley-prism-based imaging sensor.

In order to analyze the imaging characteristics of the Risley prism, an inverse ray tracing model of the system [28] is established based on the theoretical model of Figure 1, as shown in Figure 2, which describes in detail the deflection process of the light beam passing through the Risley prisms. The center of the circle on the outer surface of the camera lens is set as the origin, and a Cartesian coordinate system is established. The four surfaces of the prism are labeled as 1, 2, 3, and 4 in the direction away from the camera lens, and the unit normal vectors of the four surfaces are ${\hat{N}}_{1},{\hat{N}}_{2},{\hat{N}}_{3},$ and ${\hat{N}}_{4}$, which can be expressed as:

(2) $${\hat{N}}_{1}=[\cos(\theta_{1})\sin(\alpha ),\sin(\theta_{1})\sin(\alpha ),\cos(\alpha )],$$

(3) $${\hat{N}}_{2}=[0,0,1],$$

(4) $${\hat{N}}_{3}=[0,0,1],$$

(5) $${\hat{N}}_{4}=[-\cos(\theta_{2})\sin(\alpha ),-\sin(\theta_{2})\sin(\alpha ),\cos(\alpha )],$$

where $\alpha =\alpha_{1}=\alpha_{2}$. The incident light vector $\hat{S}=[x,y,z{]}^{T}$ of the Risley prism is unitized to obtain the unit incident light vector ${\hat{S}}_{0}=[x_{0},y_{0},z_{0}{]}^{T}$, and the beam vector after refraction through the four prism faces is defined as ${\hat{S}}_{i}=[x_{i},y_{i},z_{i}{]}^{T},i=1,2,3,4$, which can be calculated from Snell’s law [36] in vector form as:

(6) $${\hat{S}}_{i}=\frac{n_{i-1}}{n_{i}}[{\hat{S}}_{i-1}-({\hat{S}}_{i-1}\cdot {\hat{N}}_{i}){\hat{N}}_{i}]+{\hat{N}}_{i}\sqrt{1-{\left(\frac{n_{i-1}}{n_{i}}\right)}^{2}+{\left(\frac{n_{i-1}}{n_{i}}\right)}^{2}{\left({\hat{S}}_{i-1}\cdot {\hat{N}}_{i}\right)}^{2}},$$

where *n_i_*_−1_ denotes the refractive index of the medium at the incident end of the prism, *n_i_* denotes the refractive index of the medium at the exit end of the prism, and the refractive index in the air is usually taken as 1.

Through Equations (1) to (5), the refracted beam ${\hat{S}}_{1},{\hat{S}}_{2},{\hat{S}}_{3},{\hat{S}}_{4}$ can be calculated step by step, and finally, using the emitted ray vector ${\hat{S}}_{4}=[x_{4},y_{4},z_{4}{]}^{T}$, the deflection angle *Φ* and azimuth angle *Θ* of the final emitted ray are calculated as:

(7) $$\left\{ \begin{array}{l} \Phi =\arctan(\frac{\sqrt{x_{4}{}^{2}+y_{4}{}^{2}}}{z_{4}})\\ \Theta =\arctan(\frac{y_{4}}{x_{4}}) \end{array}\right..$$

The value of *Θ* is in the range of [0, 2π], so it is necessary to determine the specific value of *Θ* according to the positive and negative signs of $x_{4}$ and $y_{4}$. Define the object distance as *L*, and define the coordinates of the intersection of the incident ray of the system with the object plane as $P_{r}\left(x_{r},y_{r}\right)$. According to the similar triangle principle, $x_{r}$ and $y_{r}$ can be expressed as:

(8) $$\left\{ \begin{array}{l} x_{r}=(\frac{x_{4}}{z_{4}})\cdot L\\ y_{r}=(\frac{y_{4}}{z_{4}})\cdot L \end{array}\right..$$

### 2.2. Image Pre-Processing Methods

Light rays with different angles of incidence will produce outgoing rays with different angles of deflection when refracted by a prism. The change in deflection angle has a nonlinear trend, which will lead to a nonlinear change in the imaging FOV as well as resolution. The expanded FOV has the problem of resolution reduction in special areas, such as edges, which will have a greater impact on the calibration of the camera. The thick ends of the two prisms are connected to their axes, and the lines are recorded as *l*_1_ and *l*_2_, and the angle between *l*_1_ and *l*_2_ is defined as the relative angle of rotation of the Risley-prism-based imaging sensor. When the relative angle of the two prisms is large, part of the original FOV will be deflected into an irregular shape, while the resolution decreases along the direction of the prism angle, and the pixel points in the imaging area are nonlinearly distributed. In this case, when acquiring images with different spatial distributions, slight camera shake and poor spatial light can cause blurring of the image in a specific area and affect the calibration accuracy.

The above problem is due to the refraction of the prism; some parts of the imaging area are reduced in resolution, resulting in the blurred edges of the tessellated images taken in this area, and the corner points cannot be recognized correctly. In order to improve the success rate of corner point recognition, the bicubic interpolation algorithm [37] is used to pre-process the more blurred images by adding pixels to improve the sharpness of the image edges while ensuring the edge features. The principle is to use the gray values of 16 points around the point to be sampled for three times interpolation, taking into account not only the gray effects of the four directly adjacent points but also the effects of the gray value change rate between each neighboring point, and its basic function form is as follows:

(9) $$W\left(x\right)=\{\begin{array}{ll} \left(a+2\right){\left|x\right|}^{3}-\left(a+3\right){\left|x\right|}^{2}+1, & \left|x\right|\leq 1\\ a{\left|x\right|}^{3}-5a{\left|x\right|}^{2}+8a\left|x\right|-4a, & 1<\left|x\right|<2\\ 0, & 2<\left|x\right| \end{array},$$

where a=1. The grayscale value of the pixel (x,y) to be found is calculated as:

(10) $$f(x,y)=f(i+u,j+v)=MQN^{T},$$

where *u* and *v* are the distances between the pixel (x,y) and the original pixel (i,j) in the *x* and *y* directions, respectively, and

(11) M=[W(1+v) W(v) W(1−v) W(2−v)],

(12) N=[W(1+u) W(u) W(1−u) W(2−u)],

(13) $$Q=\left[\begin{array}{llll} f(i-1,j-1) & f(i-1,j) & f(i-1,j+1) & f(i-1,j+2)\\ f(i,j-1) & f(i,j) & f(i,j+1) & f(i,j+2)\\ f(i+1,j-1) & f(i+1,j) & f(i+1,j+1) & f(i+1,j+2)\\ f(i+2,j-1) & f(i+2,j) & f(i+2,j+1) & f(i+2,j+2) \end{array}\right].$$

### 2.3. Distortion Correction Method

The sources of aberrations in the Risley-prism-based imaging sensor mainly include lens aberrations, dual optical axis problems, and Risley-prism imaging aberrations. Lens aberration is caused by the inability of the lens set in the camera to reach the ideal state in processing and assembly, which is small but not negligible. The dual-optical axis problem includes the optical axis parallelism problem and the parallax problem that exists when imaging the Risley-prism system. When processing and installing the equipment to correct the two optical axes, adjusting the aperture spacing of the dual prism system can effectively reduce the impact of the dual optical axis problem; the aberration effect can be ignored. The imaging aberration brought by the Risley prisms is caused by the nonlinear deflection of the beam by the wedge prism, and the impact of this aberration is much larger than the first two, which is the main source of the aberration of the Risley-prism-based imaging sensor.

The imaging principle of the Risley-prism-based imaging sensor shows that when the prisms are rotated around the axis, different FOV deflection directions cause different imaging aberrations, i.e., the Risley-prism-based imaging sensor introduces a dynamic variable-parameter imaging aberration. For this variable-parameter imaging aberration, it is not enough to describe the internal and external reference matrices using only a linear model, which is described here in combination with a nonlinear model, which can be expressed as:

(14) $$\left\{ \begin{array}{l} \bar{x}=x+\psi_{x}\left(x,y\right)\\ \bar{y}=y+\psi_{y}\left(x,y\right) \end{array}\right.,$$

where $\left(\bar{x},\bar{y}\right)$ is the ideal image plane coordinate without aberration, $\left(x,y\right)$ is the actual image plane coordinate, $\psi_{x}\left(x,y\right)$ is the total nonlinear aberration in the *x*-direction, $\psi_{y}\left(x,y\right)$ is the total nonlinear aberration in the *y*-direction, and the two aberration values can be described by the following equations:

(15) $$\left\{ \begin{array}{l} \psi_{x}\left(x,y\right)=x+x\left(k_{1}r^{2}+k_{2}r^{4}+k_{3}r^{6}\right)+\left[2p_{1}xy+p_{2}(r^{2}+2x^{2})\right]+\omega_{1}r^{2}\\ \psi_{y}\left(x,y\right)=y+y\left(k_{1}r^{2}+k_{2}r^{4}+k_{3}r^{6}\right)+\left[2p_{2}xy+p_{1}(r^{2}+2y^{2})\right]+\omega_{2}r^{2} \end{array}\right.,$$

where $r=\sqrt{x^{2}+y^{2}}$; $k_{1},k_{2}$, and $k_{3}$ are the radial distortion coefficients; $p_{1}\;\mathrm{and}\;p_{2}$ are tangential distortion coefficients; $\omega_{1},\omega_{2},\;and\;\omega_{3}$ are thin prism distortion coefficients.

For the aberrations of dynamic variable parameters brought about by the Risley-prism-based imaging sensor, this paper, on the basis of obtaining the inner and outer parameter matrices and radial aberration coefficients using the existing mature calibration method [38,39], adds thin prism aberrations and tangential aberration coefficients for calculation, and nonlinearly optimizes the parameters using the Levenberg–Marquardt algorithm [30]. The Levenberg–Marquardt algorithm minimizes the objective function by iteration, and the minimization objective function can be expressed as:

(16) $$\begin{array}{l} F(X)=\sum_{i=1}^{n}f(M_{i},k_{1},k_{2},k_{3},p_{1},p_{2},\omega_{1},\omega_{2},R_{i},t_{i})\\ =\sum_{i=1}^{n}{\left(\alpha_{x}{\bar{x}}_{d,i}+x_{0}-x_{i}\right)}^{2}+{\left(\alpha_{y}{\bar{y}}_{d,i}+y_{0}-y_{i}\right)}^{2} \end{array},$$

where $M_{i},R_{i},\;and\;t_{i}$ are the parameters calculated using the linear model; $\alpha_{x}$ and $\alpha_{y}$ are the scale factors of the axes; $\left(x_{0},y_{0}\right)$ is the position of the main point of the image plane; $\left({\bar{x}}_{d,i},{\bar{y}}_{d,i}\right)$ and $\left(x_{i},y_{i}\right)$ are the theoretical and actual coordinates of the model for the *i*th point, respectively.

## 3. Design of the Risley-Prism-Based Imaging Sensor

### 3.1. Architectural Design of the Risley-Prism-Based Imaging Sensor

**Figure 3:**
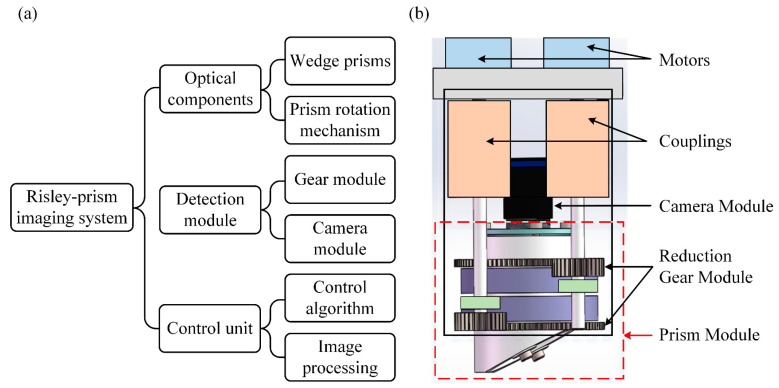
Design of the Risley-prism-based imaging sensor: (**a**) system components; (**b**) 3D diagram of the sensor.

The Risley-prism-based imaging sensor is a typical optomechanical pointing system, which involves the integration of several subsystems, mainly the image recognition system, the beam pointing model, and the prism control system. The Risley-prism-based imaging sensor has high stability and high precision optical imaging performance, which generally consists of an optical receiving element, detection module, and control unit [40], as shown in Figure 3a. The optical element module is mainly designed to change the pointing position of the outgoing beam to achieve spatial orientation scanning and determine the FOV range where the target is located. The module is composed of optics, mainly two wedge-shaped prisms placed co-axially in an integrated mirror holder. The detection module includes a gear transmission group and a camera module. The gear transmission module contains motors, couplings, and reduction gears that drive the rotation of the prism frame; the camera module is mainly a camera and is mounted co-axially with the prism group; the main function of the detection module is to change the relative rotation angle between the prisms to achieve the visual axis scanning and pointing, and the specific structure is shown in Figure 3b. The decision-making unit of the whole system is the system control chip, which mainly deals with two core matters: controlling the rotation of two motors and collecting and processing image information.

### 3.2. Control System Design of the Risley-Prism-Based Imaging Sensor

**Figure 4:**
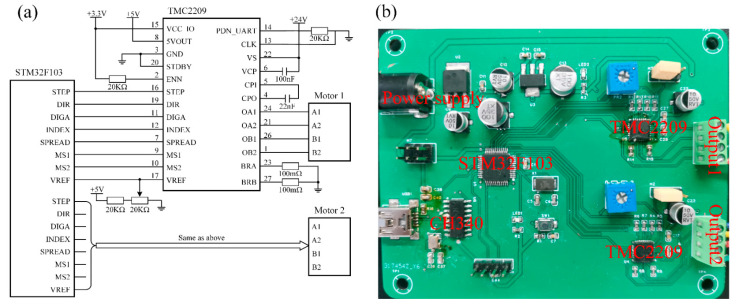
Motor control circuit: (**a**) circuit schematic; (**b**) printed circuit board.

In order to realize the angle control of the prisms, a motor control circuit is designed as shown in Figure 4, where (a) represents the schematic diagram and (b) is the printed circuit board. The STM32f1 series chip is selected as the main control unit, and the Trinamic TMC2209 chip is selected as the driver of the motor. The computer communicates with the control unit through serial communication, thus realizing the control of two motors.

**Figure 5:**
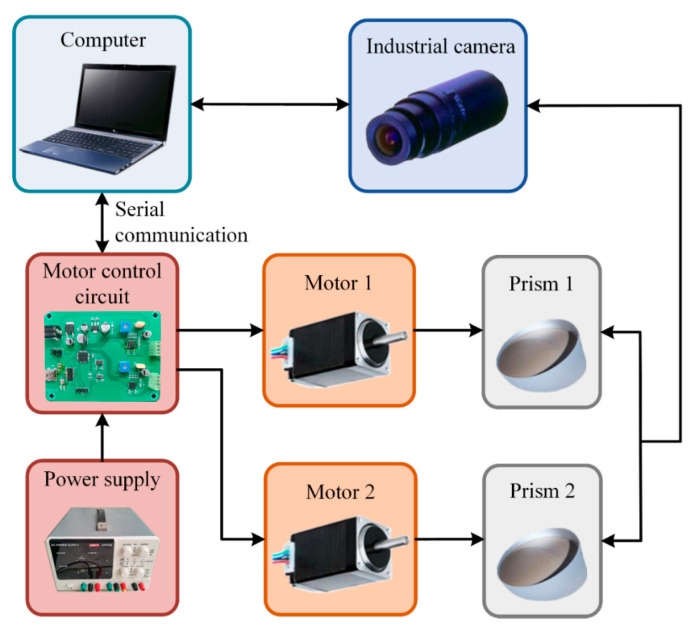
Control system architecture of the Risley-prism-based imaging sensor.

The control system design of the Risley-prism-based imaging sensor is shown in Figure 5, which mainly consists of a computer, a motor control circuit, a power supply, two motors, two prisms, and an industrial camera. The computer sends commands to the motor control circuit through serial communication to drive the motor to rotate, which makes the prisms deflect from each other, thus driving the camera FOV to move. The industrial camera is used to acquire images.

A pair of round wedge prisms are used, and the specific parameters are shown in Table 1. The industrial camera adopts the Watec CCD camera (model wat-704r) with a resolution of 640 × 480 pixels and an FOV of 51° × 40°. The motor is a two-phase, four-wire hybrid stepper motor (model YIXING 20BYGH300) with a driving voltage of 3.9 V.

## 4. Automatic Calibration Method for the Risley-Prism-Based Imaging Sensor Based on the Two-Axis Turntable

The two-dimensional calibration of the camera usually adopts the method of detecting the feature points of the calibration plate, then establishing the single response matrix, solving the linear system, and performing optimization to obtain the internal and external parameters of the camera. A chessboard is great for calibration because it is a regular, high-contrast pattern, which makes it easy to detect automatically. The camera calibration method from Zhang, Z. [38] uses a chessboard for calibration, which is the most representative calibration method at present, but the method usually requires manual movement of the calibration plate during the calibration process so that different angles can be taken, which also affects the stability and matching accuracy of the camera calibration to a certain extent. An automatic calibration method based on a two-axis turntable is designed to achieve rapid automated calibration of the Risley-prism-based imaging sensor.

### 4.1. The Traditional Calibration Method

**Figure 6:**
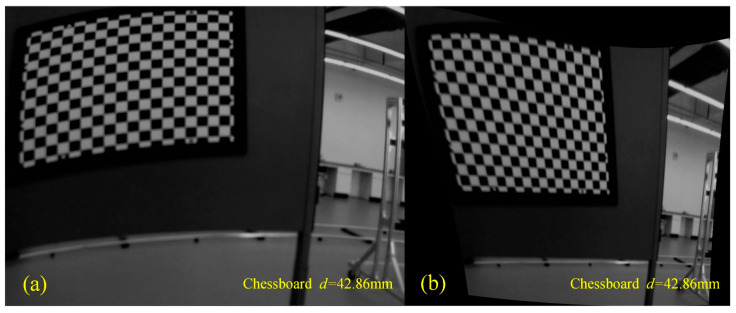
Comparison before and after correction with manual moving calibration plate: (**a**) uncorrected images and (**b**) corrected images.

During the calibration process of the manual moving calibration plate method, we found that the accuracy of the calibrated reprojection was extremely unstable when the image of the tessellated calibration plate was taken randomly. In addition, the FOV expanded by the prism is irregularly quadrilateral, and the pixel distribution in the FOV area is no longer uniform. When the calibration board is manually moved so that it is at the corners of FOV, corner distortion often occurs when the image is corrected with the calibrated camera parameters, as shown in Figure 6, where *d* represents the length of a chessboard grid.

### 4.2. Two-Axis Turntable-Based Automatic Calibration Method

**Figure 7:**
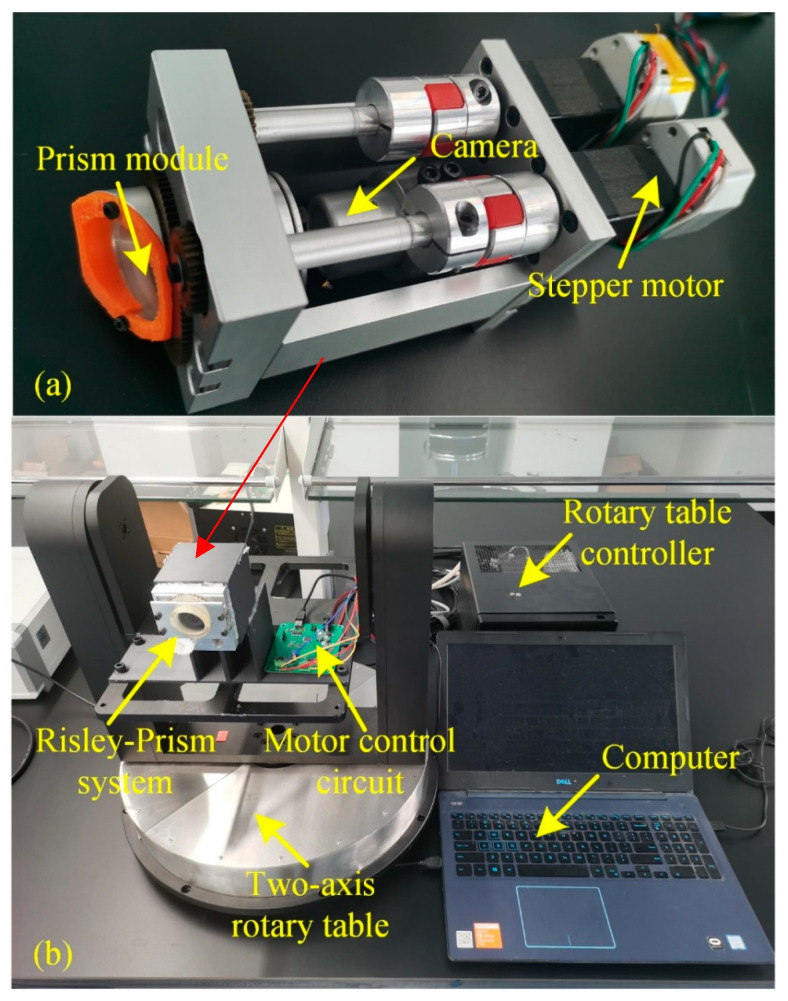
Experimental setup: (**a**) Risley-prism-based imaging sensor; (**b**) automatic calibration system.

The physical diagram of the Risley-prism-based imaging sensor designed is shown in Figure 7a, whose mechanical parts are produced by precision five-axis machine tools to greatly ensure the co-axially between the camera view axis and the prism optical axis. The combination of precision motion control technology and software algorithms can realize the control of the prism’s rotation angle easily and with high accuracy.

Considering the practical application of this system, a specific prism rotation angle is generally selected for calibration and encapsulated into a database [25], which not only requires taking a large amount of image data but also has higher requirements for the stability and accuracy of each captured image. In order to meet the above requirements, an automatic calibration method is designed, which requires the Risley-prism-based imaging sensor mounted on a precision two-axis turntable, as shown in Figure 7b. Then, the Risley prism motor control, the two-axis turntable control, and the camera imaging are integrated into a host computer program to precisely control the deflection angle of the prism and the rotation angle of the two-axis turntable.

**Figure 8:**
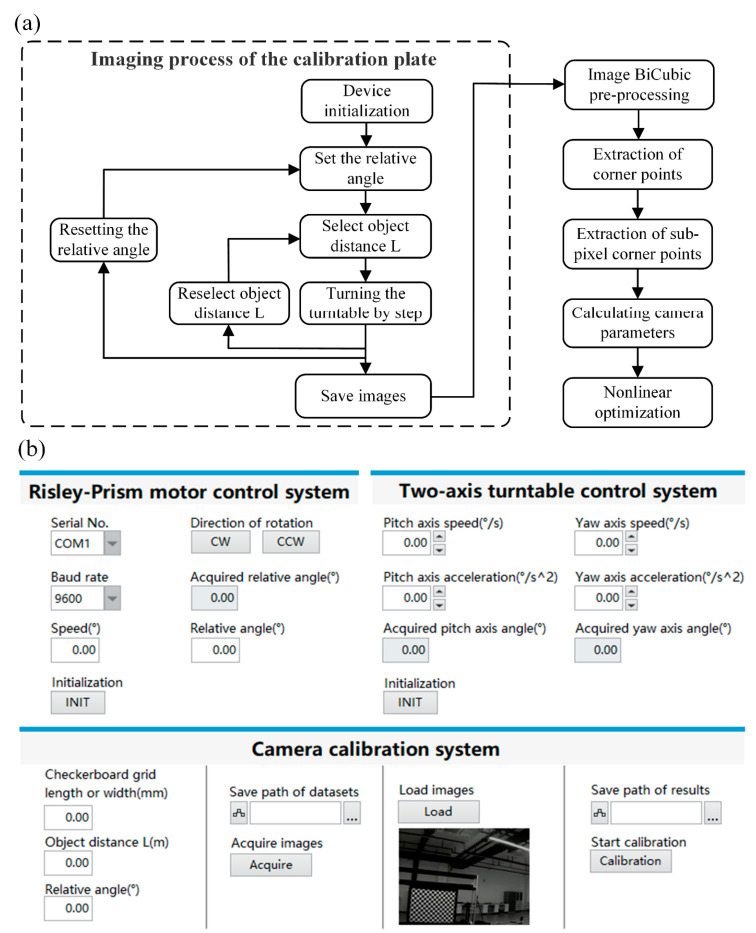
Flowchart and program: (**a**) the overall flowchart of the automatic calibration method; (**b**) the user interface of the calibration program.

The overall flow diagram of the calibration method is illustrated in Figure 8a, and the user interface is shown in Figure 8b. The process of quickly obtaining the calibration plate image dataset: first, before calibration, the camera bracket needs to be adjusted to make the camera view axis coincide with the rotation center of the two-axis turntable and initialize the system; then, set the relative rotation angle of the Risley prisms and the distance *L* between the calibration plate and the camera; next, adjust the angle of the turntable so that the calibration plate is located at the top left and bottom right of the image in the camera, respectively, and record the *X*- and *Y*-axis rotation positions of the turntable for both cases; in the next step, select the appropriate rotation step in *X* and *Y* directions, take one picture per rotation step, and then take a picture of the calibration plate evenly distributed in FOV of the camera and save it; finally, reselect the relative angle of the Risley prisms and the distance *L*, and repeat the above steps.

The essence of aberration correction is to transform the pixel points in the image one by one through the nonlinear model in Equation (12). The steps of image aberration correction for the calibration method are as follows: the first step is to pre-process the calibration plate image dataset obtained from the above shots by bicubic interpolation; the second step is to extract the detected corner point information of each image; the third step further extracts its sub-pixel corner point information; the fourth step uses these corner point information to find out the internal and external parameters of the camera and the actual radial aberration coefficient; the fifth step uses the Levenberg–Marquardt algorithm to nonlinearly optimize the parameters.

## 5. Experimental Validation

In order to verify the automatic calibration method, the wedge angle of the prism in the experimental system is $\alpha_{1}=\alpha_{2}=14.85{}^{\circ}$, the refractive index is $n_{1}=n_{2}=1.515$, the camera resolution is 640 pixel × 480 pixel, the camera FOV is 51° × 40°, the accuracy of the two-axis turntable is ±0.05°, the effective size of the checkerboard grid is 771.48 mm × 771.48 mm, and its square grid size is 42.86 mm × 42.86 mm.

The reprojection error is an important indicator in evaluating the calibration accuracy of the camera, and a smaller reprojection error represents a higher aberration correction accuracy. The resulting reprojection errors are compared as follows:

From Table 2, the further the checkerboard calibration plate is from the camera, the smaller the reprojection error is. When the distance is 0°, the average reprojection error reaches 0.11 pixels, and the average reprojection errors of 90° and 180° are also around 0.25 pixels, which are relatively low and prove the reliability of the calibration method.

**Figure 9:**
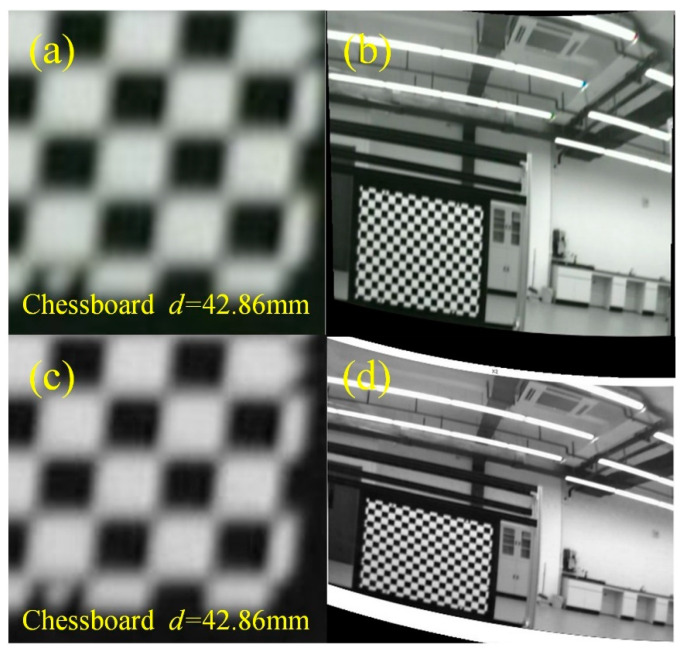
Image comparison before and after pre-processing. (**a**) Failed image of corner point detection; (**b**) successful image of corner point detection after pre-processing; (**c**) correction image before pre-processing; (**d**) correction image after pre-processing.

Figure 9 shows the comparison images before and after pre-processing with the bicubic interpolation algorithm. It can be seen that when the distance between the calibration plate and the camera is far, and due to the dispersion caused by the Risley prism, the corner points are blurred when imaging, which leads to the error of the later algorithm in recognizing the corner points, while the corner points can be recognized normally after the pre-processing of the image with the bicubic interpolation algorithm. The effect of taking the pre-processed calibration plate image for distortion correction at the same time is shown in Figure 9d, and the correction effect is better, which indicates that the bicubic interpolation has a better effect of increasing pixels.

**Figure 10:**
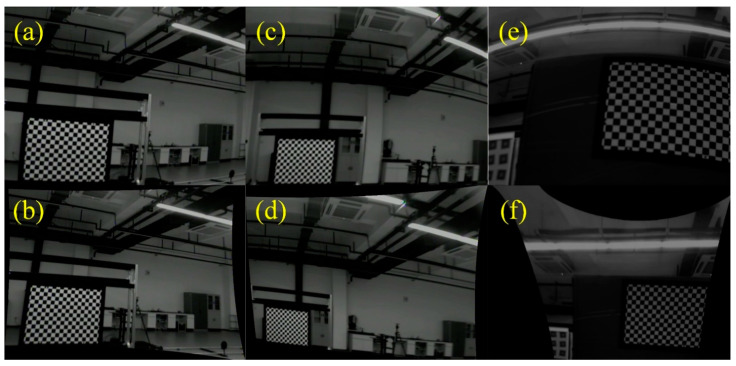
Images before and after correction: (**a**,**b**) deflection angle 0°; (**c**,**d**) deflection angle 90°; (**e**,**f**) deflection angle 180°.

The results of the calibration of the aberrated images using the camera parameters obtained by the bicubic interpolation algorithm and the calibration method designed are shown in Figure 10. The aberrations in (b), (d), and (f) are significantly reduced, which proves that the proposed method can effectively correct the imaging aberrations of the Risley-prism-based imaging sensor. In summary, the calibration method of the Risley-prism-based imaging sensor based on the two-axis turntable processed by the bicubic interpolation algorithm has the characteristics of rapidity and automation, and the calibration is more accurate and reliable.

## 6. Conclusions

In order to improve the calibration accuracy, speed, and stability of the Risley-prism-based imaging sensor, an automatic calibration method is proposed based on a two-axis turntable. The technical route of this paper mainly includes three parts: (1) analyzing the deflection relationship between incoming lights and outgoing lights by establishing a beam propagation model of the Risley-prism-based imaging sensor and building a suitable imaging aberration model for the imaging aberration of the system by combining the nonlinear model; (2) aiming at the problems of a large amount of image data acquisition, a large amount of calculation, and unstable calibration accuracy in the calculation process of the Risley-prism-based imaging sensor, a method based on the two-axis turntable is proposed to realize the automatic calibration of the system; (3) addressing the problem of unrecognizable corner points due to blurring caused by excessive imaging aberration in the calibration process, a pre-processing method is proposed to improve the resolution of the image by using the bicubic interpolation algorithm. Experimental results proved that the method is more convenient and automated, and the calibration results are more reliable.

Because of the dynamic change in the imaging aberration correction parameters of the Risley-prism-based imaging sensor, its calibration needs to be calibrated separately for different rotation angles of the Risley prisms, which is a complicated and time-consuming process, and the automated calibration method proposed in this paper can solve this problem well. In Risley-prism-based applications such as microscopic observation and robotic vision, the method proposed in this paper accelerates the construction of the system and improves accuracy. The future development of the method includes: (1) it has only been verified in a single Risley-prism-based imaging sensor, and it can be verified in the combination of multiple Risley-prisms imaging sensors in the future; (2) image stitching and image fusion techniques can be used to expand the whole FOV for the completed Risley-prism-based imaging sensor; (3) the stitched large FOV image stream can be used to realize the display of large FOV video effect; (4) comparative experimental validation of the auto-calibration method in this paper against related methods.

## Figures and Tables

**Table 1:** Prism parameters.

Prism Parameters	Value
Diameter	25 mm (maximum error 0.10 mm)
Refractive index	1.515
Wedge angle	14°51′0″ ± 0°0′30″
Thickness	3.00 ± 0.20 mm

**Table 2:** Reprojection error for different *L* and relative rotation angles of the Risley prisms.

*L* (m)	Reprojection Error (pixel)		
	0°	90°	180°
1.2	0.45	1.40	1.83
2.4	0.19	0.79	0.38
3.6	0.11	0.25	0.26

## Data Availability

The data that support the findings of this study are available from the corresponding author, F.H., upon reasonable request.
